# On the Mimoses; or, a Descriptive, Diagnostic, and Practical Essay, on the Affections Usually Denominated Dyspeptic, Hypochondriac, Bilious, Nervous, Chlorotic, Hysteric, Spasmodic, &c.

**Published:** 1820-04-01

**Authors:** 


*On the Mimoses; or, a descriptive, diagnostic, and practical Essay, on the Affections usually denominated Dyspeptic, Hypochondriac, Bilious, Nervous, Chlorotic, Hysteric, Spasmodic, &c.* By MARSHALL HALL, M. D. Author of a Treatise on Diagnosis, &c. 8vo. p. 176, London.

OF all the diseases incident to humanity, there are none perhaps more prevalent or distressing, none more intractable than those which form the subject of this Essay. Every attempt, therefore, to elucidate their nature, or improve their method of treatment, is no less deserving the approbation of the philanthropist, than the thanks of the physician. On this account we feel indebted to Dr. Hall for the little volume before us. But, whilst we are unwilling to withhold from him the meed of applause due to his well-intended endeavours, and evident talent, duty compels us to acknowledge, that, in our opinion, they have not been crowned with all the success which investigations instituted for so laudable a purpose, and conducted with so much zeal and labour, deserved. Laying aside, however, any further prefatory observations, we proceed to present the reader with an outline of the Treatise itself; after which, we shall consider in detail such parts of it as appear to us more particularly worthy of remark.

There is then, as Dr. Hall truly observes, a class of diseases usually called dyspeptic, hypochondriac, bilious, nervous, &c. These he proposes to designate by the title " Mimoses" ; of which he makes five species.

1st. The mimosis acuta.

2d. The mimosis chronica; or dsypepsia with hypochondriasis.

3d. The mimosis de color; or chlorosis.

4th. The mimosis urgens ; or hysteria.

5th. The mimosis inquieta.

Previously, however, to entering upon a description of these several species, Dr. H. logically enough, presents us with the definition of his generic term. " The mimoses, (says he) are characterized by being complex, multiform, various and changeable, and by imitating, *from the appearance and predominance of particular symptoms in particular instances,* other diseases very different in their nature." Now, it either is, or ought to be, the object of every definition, to convey to the mind clear and distinct ideas of the thing signified. But that object has been greatly disregarded in the case before us. Under so great a number of words is our author's meaning buried, that it requires much painful search, before we can come at any tolerable conjecture as to what he would really wish to communicate. In proof of our assertion, we would ask, where is the necessity of that epithet, " various ? " If the mimoses are characterized by being multiform and changeable, is not their *variety* plainly implied ? If, again, the essential quality of the mimoses be to imitate other diseases, really different in their nature, it is obvious, that such imitation can only proceed from the appearance or predominance of some symptoms common to both classes of disorders. But, objectionable as is our author's definition on account of its verbosity, it is still more so on the ground of its inconsistency. For, according to one of the plainest and most universally received rules of logic, whatever enters into the definition of the genus must be applicable to all the species comprehended under it. Not so, however, with the dialectics of Dr. Hall. In his definition of a genus of disease, to wit, the mimoses, *particular* symptoms are said to occur in *particular* instances; or, in other words, such symptoms are referred to *some* species of that genus, and not to *others*; a circumstance wholly irreconcilable with the nature of a general definition. The paragraph immediately following it, contains a remark with respect to the causes of this class of diseases, so contrary to our own experienced conviction, that we cannot refrain from animadverting upon it a little particularly. The passage to which we refer, is, where Dr. H. asserts, that the diseases under consideration, " have been too exclusively attributed to a deranged state of the chylopoietic viscera." Now, if there be one truth in medicine more susceptible of proof than any other, it is the very converse of this proposition. In the whole catalogue of writers on dyspeptic, hypochondriac, and other nervous complaints, there is not, we will venture to assert, one to be found, chargeable with ascribing to the chylopoietic viscera a greater share in the production of these disorders than really belongs to them. On the contrary, the great and glaring error has been to ascribe infinitely too little. The deranged states of the chylopoietic viscera (in our judgement the chief source of the diseases in question) they, with a very few exceptions, have almost entirely overlooked; witness their general silence on the subject of these morbid conditions, and that farrago of heating, stimulant, and antispasmodic remedies so commonly, but uselessly employed. Amidst all these misconceptions, however, some writers there are, who, in our opinion, seem to have entertained surprizingly just notions, both as to the pathology and treatment of nervous disorders.

The celebrated Dr. Cheyne, whose character as a philosopher, as well as a physician, must ever retain the most exalted station, declared nearly a century ago, that " the VAPOURS are the first symptoms of a real chronical disease, which, if neglected, will end in the spoiling of some great bowel;" and in another place, he proposes to call such affections *glandular*, on account of the inseparable connexion conceived to subsist between them and the liver, spleen, or some other great and important viscus. In short, so strongly was he impressed with the truth of this doctrine, that it is perpetually recurring throughout his valuable Treatise on the English malady. Agreeably then to such a view of his subject, he substituted for the common method a plan of treatment by mercurials, evacuants, and bitter tonics; and, if it be fair to judge of the success by the extent of his practice in this particular department, it will afford a very weighty argument in favour of the justness of his conclusions. The illustrious Commentator on Boerhaave also, in reference to such disorders as now occupy our attention, ascribes them to the stagnation and collection of fluids in the hypochondriac vicera, as the spleen, &c. which vitiating the secretions of these glands, deranges the first digestion, and thus produces the long train of nervous symptoms.* And, to pass on to the writers of our own times, who labour in the field of medicine with so much assiduity and success, it is impossible not to perceive a striking coincidence in the opinions of some modern physicians, and of those great, but more ancient authorities just cited. In some works, recently published, the doctrines of Cheyne and Van-Swieten have been explained with such perspicuity, so ingeniously extended, and confirmed with such a force of argument, as to threaten the total overthrow of those errors, which to the retardation of science, and the bane of mankind, have been long suffered to regulate the treatment of nervous disorders. Should, however, Dr. H. notwithstanding these strong testimonies, still consider his position tenable, we beg to refer him to the dissections of Morgagni, where he will discover abundant proofs of its insufficiency.†

But it is now time that we enter upon the consideration of the various species of the Mimoses. Following, therefore, the order in which they are set down, we begin with the Mimosis Acuta. A large portion of the Treatise before us, is dedicated to this species of the disease, which Dr. H. appears to consider as hitherto undescribed. In our opinion, however, it is synonimous with, and substantially the same disease as the common dyspepsia of all medical writers. The very definition of this species, as

* Vide Van Swieten's Comment. Vol. iii. page 482 and 3, 4to. Edition.

† Amongst other examples, vide Epist. 37, Art. 2, 17, 23, 29; Epist 5, Art. 19; and Epist. 39, Art. 1, and 4.

given by Dr. H. is a sufficient proof of the truth of our assertion; but yet the further history of it, together with his minute detail of cases, serves to put the matter beyond all doubt.

"The mimosis acuta (says Dr. H.) is characterized by weakness, tremor, fluttering, faintishness, tendency to perspiration, susceptibility to hurry and agitation, and loss of flesh."

He then divides it into a severer and less severe form. In illustration of the former he gives five cases, in every one of which there is the clearest evidence of disordered digestion. Thus, in the 1st case we are told, there was pain in the hypochandriac region. In the 2d, costiveness, with offensive and scanty stools. In the 3d, constipation, loaded tongue, fluttering, vertigo, head-ache, and afterwards diarrhœa. In the 4th, oppression and fluttering at the stomach, with flatulence, eructation, head-ache, nausea, and vomiting. And, in the 5th, and last case, fluttering at stomach, constipation, head-ache, anorexia, and eructation.

Of the less severe form of the mimosis acuta, Dr. H. favours us with only two cases, in which, if possible, the characters of dyspepsia are yet more strongly marked. To this list he subjoins three other cases, with a view of illustrating that part of his general definition, upon which we have offered our sentiments pretty fully. These were instances, in which the predominant symptom caused them to be mistaken for diseases of the heart. But here also, it would be no difficult task to trace out the most satisfactory marks of derangement in the chylopoietic function. With all the above evidence before our author, we cannot comprehend how he could fail to recognize the identity of the mimosis acuta, with the affection usually denominated dyspepsia.

But to proceed. Having dispatched the descriptive part of this new disease, Dr. H. enters upon his favourite subject of diagnosis; and here indeed we cannot but lament the misapplication of Dr. H's unquestionable talent for discrimination, when we observe three entire pages of his work occupied with a recital of the distinctive characters between the mimosis acuta, and the several species of acute, slow, and malignant fevers. For so obviously dissimilar, appear to us the complexions of these disorders, that we cannot imagine them capable of being confounded by any class of medical practitioners.

Dr. Hall next points out numerous marks of discrimination between the milder form of the "mimosis acuta,"

and insidious organic diseases, strumous affections of the mesentery in adults, and the effects of drunkenness. In our apprehension, however, these efforts at distinction are more exceptionable in their result than the former, because it is their manifest purpose to constitute a difference betwixt diseases, whose identity we conceived to be one of the best established truths in medical science.

Passing over these diagnostics, however, we are led to the consideration of what Dr. H. denominates "THE COMPLICATION OF THE MIMOSIS ACUTA WITH OTHER DISORDERS;" a subject which is, in very truth, what it professes to be, abundantly complicated. Of these complications, Dr. H. makes no less than ten kinds.

1. With diseases of the brain; 2. phthisis pulmonalis; 3. paroxysms of dyspnœa; 4. disease of the heart; 5. organic diseases of the stomach; 6. convulsions, and spasmodic affections; 7. pain in the epigastric, hypochondriac, and chondiliac regions; 8. symptoms of diseased bladder; 9. melæna; and, 10. icterus. To each of these instances of complication, cases are added by way of illustration; and, bearing in mind our notion of the identity of the mimosis acuta with the disease usually denominated dyspepsia, we have no hesitation in admitting their existence to the utmost extent of Dr. H's wishes: and with this impression on our minds, we pass on to the next chapter, which treats of the *causes and remedies of the mimosis acuta.* In relation to the former of these topics, are enumerated watching, fatigue, anxiety, errors in diet, impure air, filthiness, and above all, sedentary habits. So strictly true do we consider this ætiological statement, and at the same time so conformable to the convictions of every unprejudiced and observing mind, that we cannot but regard all remarks in confirmation as wholly unnecessary. Neither are we more at variance with the *plan of treatment* immediately subjoined; which, it appears, consisted in the free use of laxative medicines, a light nutritious diet, exercise in the open air, and the cold bath. This, it would seem, is the very method long ago adopted by Dr. Cheyne, of evacuants and tonics: at any rate, it is one, we will venture to assert, which rests on too firm a basis of argument and observation, ever to be endangered by the utmost efforts of sophistry or error. On the contrary, it must, we are persuaded, acquire strength, in proportion to the extent of its application, and ultimately overthrow that practice of prescribing stimulants, which has so long obstructed the progress of our art, and added to the sum of human misery.

In allusion to the laxative remedies employed by Dr. H. we must not omit to mention his habit of administering them in conjunction with mercurial preparations. When diarrhœa, with scanty, fetid, and dark-coloured stools occurred, as a symptom of the mimosis acuta, he found it yield most readily to the quicksilver pill, followed up with doses of rhubarb; which, on account of its tonic quality, he particularly recommends in such cases. In some instances of constipation, Dr. H. found it requisite to purge fully; but his general method was, to procure daily one copious and loose evacuation, until the return of the patient's health was indicated by his recovery of flesh and strength. With regard to *diet,* Dr. Hall's remarks are generally good; but we confess it startled us a little, to find him interdicting the use of *lamb,* as one of the flesh meats: nor were we less surprized at the very favourable opinion he expresses as to the advantage of drinking *malt liquor,* a beverage which, in dyspeptic complaints, is so apt to offend, as to become, with medical practitioners, the subject of general prohibition.

In all cases of mimosis acuta, complicated with other diseases, Dr. H. advises the exhibition of such remedies as are best adapted to the removal of the extraneous affection, in combination with those just proposed for the more simple form of the disorder.

Such is the substance of Dr. Hall's chapter on the mimosis acuta; from which we pass on to the consideration of his next species, to wit, the *mimosis chronica.* Under this appellation, Dr. H. comprehends the diseases usually denominated dyspepsia and hypochondriasis; and we are glad to find him admitting at the onset, the intimate alliance of these complaints with that just treated of; from which, he observes, they may originate. "The mimosis chronica (says Dr. H.) is denoted, in general, by fits of despondency and gloom, of invincible disinclination for motion; of pain about the head; sinking at the præcordia; and heat, or fullness of the stomach." From the definition here quoted, the writer has excluded almost all mention of dyspeptic symptoms, having observed, in many instances of the mimosis chronica, these characteristics only. Notwithstanding, however, the experience of Dr. H. we are still disposed to think no case of hypochondriasis ever occurred, unaccompanied by marks of indigestion; and, in confirmation of our opinion, we would cite the authority of the most celebrated modern Nosologists, as Sauvages, Linnæus, Sagar, and Cullen, in all of whose definitions, the signs of dyspepsia make a most prominent feature. The Doctor himself, indeed, in *detailing* the symptoms of the mimosis chronica, admits the very point for which we contend. With what propriety, then, can his definition be applied to hypochondriasis? and if not, with how much less propriety to that disease, whose very name is derived from the existence of those qualities which are here omited as unessential?

Dr. H's delineation of the symptoms of mimosis chronica is sufficiently accurate, and requires no comment. Proceed we, therefore, to his Section of the Diagnosis, which sets out with the necessity of distinguishing the acute from the chronic species of the mimosis. This is a sentiment to which we cannot easily subscribe; and even the Doctor himself admits a close alliance between the two disorders; that they arise from the same causes; and require a similar method of treatment. Upon his own admissions, then, there can surely be no great reason for creating such a distinction. We object to it, however, from our thorough conviction of the identity of the two diseases. In truth, they appear to us and others, to be merely different stages of the same complaint;—in other words, we consider the mimosis acuta as the first effect of disordered function in the chylopoietic viscera, which, if suffered to continue, will ultimately produce such derangement in their structure, as shall constitute the very essence of the mimosis chronica. Betwixt this disease, however, and certain obscure organic affections, there is considerable ambiguity; and hence some method of discrimination becomes a desideratum. This subject, we think Dr. H. has handled with great ability. In the *treatment* of the mimosis chronica, there is nothing to arrest our attention, save that greater perseverance in the remedies proposed for the acute species, is enjoined.

We are now brought to the consideration of Dr. H's *third species of the mimoses,* to which he has annexed the epithet *decolor.* This name is synonymous with the chlorosis of other writers. After giving an excellent definition of the disease, our author proceeds to a very minute description of its symptoms; which, being consonant with what is to be met with in most treatises on the same subject, renders all remarks, on our part, superfluous. It is only necessary to observe, that this species of mimosis, like those already noticed, may assume a complicated appearance. Of the simple species, three cases are subjoined by way of illustration, and all of them were cured by a course of rhubarb or aloetic purgatives, assisted by a light diet, and regular bodily exercise: then follow three examples of the complications, two of the disorder in its inveterate stage, and finally, five instances of the chronic form of the complaint; in all of which, a similar curative process proved effectual. The descriptive part being at length discussed, Dr. H. advances towards the diagnostic; on which, with his usual partiality, he expends fifteen pages of his Essay. He is, it would seem, very fearful, lest the mimosis decolor should be mistaken either for the sudden, or the insidious forms of obscure organic disease; nor can he deny the ambiguity of these affections to be such, as to require some discriminative marks; but we, at the same time, must express our apprehensions, that many of those hair-breadth distinctions, upon which Dr. H. has so minutely dwelt, will serve little other end than that of fatiguing the reader's patience, without leaving any permanent and useful impression on his memory.

Amongst the *causes* of mimosis decolor, our author enumerates "confinement, too long lactation, frequent hæmorrhages, anxiety, fatigue, and loss of rest." But sedentary habits, as he well observes, are a far more powerful cause than any of these; and with respect to the plan of treating this disorder, it is the same as that proposed in the species, of which we have already given some account.

In his fourth chapter, Dr. H. treats of the MIMOSIS URGENS or HYSTERIA. His definition is good, and detail of symptoms full and accurate. Of all the different kinds of mimosis, this possesses the quality of imitating other disorders in the highest perfection; a list of which is given, together with the marks of discrimination peculiar to each. And here also, the causes and methodus medendi are the same as in all the other species. We have now, therefore, only to add a few words on Dr. Hall's fifth and last species. This he denominates the "MIMOSIS INQUIETA," because its leading characteristic is inquietude, restlessness, or jactitation. It is seen (says he) in some cases of dyspepsia, after inordinate evacuations, and generally as a precursor of death. From this statement, we feel disposed to regard it as an effect or symptom of some other disease, rather than as constituting any ground for a separate disorder. When it originates in dyspepsia, purgatives are advised; and in the other instances, opiates, in conjunction with such remedies as tend to allay irritation.

Having now considered, as fully as our limits will permit, the several species into which Dr. H. has divided the mimoses, we are at length brought to his concluding observations: and here we find him accounting for the great prevalence of this class of disorders in Nottingham, by a reference to the sedentary habits to which the inhabitants of that town are, by the nature of their occupations, necessarily subjected. With this opinion, our own is in the strictest conformity; and we cannot but regard it as possessing considerable interest in a physiological, as well as a pathological point of view. But, although we are willing to admit, in its most extensive application, the efficiency of the cause here assigned, we can by no means subscribe to Dr. Hall's account of its *mode of operation.*

"Sedentary habits (says our author) lessen the peristaltic motion of the intestines; and hence arise fæcal accumulations, which first disturb the functions of the bowels themselves, and afterwards those of the chylopoietic, and all the other viscera."

Now, according to our view of the subject, the order of these effects ought to be exactly reversed. The constipation and collection of stercoraceous matter, we regard as the consequence, rather than as the cause, of derangement in the functions of the secerning viscera. For the complicated structure of these organs, together with the slow and languid motion of their fluids, are facts too well known to the physiologist, to require any proof or comment. Hence, it is very reasonable to suppose their weak circulation will be more readily affected by those causes, which diminish the heart's energy, than that of other parts, thro' whose vessels the vital current flows with greater momentum and vigour. From a circulation thus enfeebled and impeded, it may fairly be presumed a corresponding change will take place in the secretions. And, if so, it is clear neither the purposes of nutrition, nor of excretion, can properly be answered. A healthy chyle will not be formed, nor the alvine contents duly expelled. Thus, then, from the disordered functions of the chylopoietic viscera, have we, by a very easy and, we trust, satisfactory method, deduced the great probability of intestinal accumulations. But, although we have advanced thus far, with the view of proving that the evil consequence of sedentary habits first affect the great glands of the body, we would by no means be understood to deny their pernicious influence over many other of the less complicated portions of the human fabric. For, by inactivity, we well know that the peristaltic motion of the intestines may soon be impaired, and in process of time, even the skin itself (as Dr. H. frequently notices) may lose its healthy colour and circulation. All we contend for is, that these are not the PRIMARY effects of sedentary habits; and consequently, that Dr. H. has erred in deducing from THEM the derangement in the functions of the chylopoietic and other viscera. There is one other point in the concluding observations, to which we would direct the attention of our readers. We allude to the apology Dr. H. makes for the use of that new word, by which his Essay is entitled. Finding, in all the disorders which he attempts to classify, a propensity to imitate other affections, he thinks this quality may be very safely assumed, as the ground-work of his generic name. Hence, he denominates the diseases in question by the appellation "Mimoses;" deriving it from the Greek μῖμος, imitator. But we are by no means disposed to regard the reasons assigned by Dr. H. in excuse of this innovation, as a sufficient plea for adding to that immense stock of technical terms, with which the science of physic is at present overburdened. For although it must be confessed, that Cullen's classification of the diseases in question is manifestly inconsistent, yet we think, had he prosecuted his inquiries a little farther, and referred to the writings of that luminary, Dr. Young, he might have obviated the "inconvenience of circumlocution," without the invention of a new name; and at the same time, have been supplied with a nosological arrangement in every way free from "hypothetical speculation." But we cannot take leave of our author, without first intreating him to bestow on any undertaking he may hereafter design for the public eye, a little more consideration than he has evinced in the Treatise, of which we have just attempted an imperfect analysis. Had he but allowed himself time for a proper and methodical arrangement of his ideas, his Essay on the Mimoses would, we doubt not, have issued from the press in a more perfect and instructive form; or, at least, it would have come into the world, divested of those glaring defects with which it at present abounds.

From this haste to come before the public, without due preparation, our author has suffered himself to be betrayed into errors, which are not merely confined to method, but which extend even to the very language of his composition. In short, a want of perspicuity, both as to thought and expression, seems to be its leading characteristic: diffuseness too, the Essay labours under in an eminent degree; and, nowhere is this fault more conspicuous than in the narration of cases, with which Dr. H. in defiance of the example of the best medical writers, has greatly augmented the size of his volume. It cannot be denied that the science of physic, like every other branch of natural philosophy, will be most successfully cultivated by the plan of induction from a long and copious list of particular instances. But, it is not therefore requisite that to all these instances, which (in professional language) are denominated cases, publicity should be given. No, it is sufficient simply to state the induction, annexing an individual case or two, as an example of all the rest.

Such was the method pursued by the great father of physic, and such also the practice of his illustrious rival in modern times, the sagacious Sydenham, whose sentiments we could wish Dr. H. had borne in mind. In allusion to this very topic, the English Hippocrates thus expresses himself: — "Unum adhuc restat de quo monendus est lector, mihi in animo non esse, sequentes paginas infinito particularium observationum numero distendere, quibus methodo ibidem traditæ fidem adstruam: frustra enim et cum tædio lectoris, repeterentur ista singulatim, quæ in summas contraxi. Satis autem habui, ad calcem cujuslibet observationis generalis, particularem hic illic adnectere, qua methodi præcedentis medulla contineretur."

In philosophizing, however, upon medical subjects, should the opposite method to the one here proposed ever come into use, we do not scruple to predict so rapid a multiplication of medical productions, as shall serve but to deter mankind from the study and cultivation of the healing art!

Were we disposed to indulge a spirit of censorious criticism, (which is at all times inconsistent with the genius and avowed object of this publication) we might point out some more imperfections in Dr. Hall's Essay; but from so unpleasant a task, we willingly abstain. We have, as we hope, faithfully, and to the best of our humble abilities, performed the duty prescribed to us; and, in so doing, we trust that the industrious Author of the Mimoses (whose talent for observation only requires a proper channel to render it honourable to himself, and useful to his brethren) will find nothing to lay to our charge as hypercritics or unreasonable censors; but, that he will peruse our animadversions with all that liberality of mind which prompted the great Cicero, when suffering reproof, to exclaim, "Non is sum qui vituperari nolim, modo vituperatio sit justa."

---

* Vide Sydenhami Opera Præfat. Versus finem.

